# Supplementary material for: Occurrence and Concentration of Chemical Additives in Consumer Products in Korea
Source: Int J Environ Res Public Health. 2019 Dec 12;16(24):5075. doi: 10.3390/ijerph16245075 (PMC6950561; doi:10.3390/ijerph16245075)
Supplement: Supplementary file 1 [file ijerph-16-05075-s001.pdf]

International Journal of
Environmental Research and Public Health

MDPI

Article

Occurrence and Concentration of Chemical Additives in Consumer Products in Korea

Syed Wasim Sardar [1], Younghun Choi [1], Naree Park [1] and Junho Jeon [1,2,*]

[1] Graduate School of FEED of Eco-Friendly, Offshore Structure, Changwon National University, Changwon, Gyeongsangnamdo 51140, Korea; syedwasim336@gmail.com (S.W.S.); dudgnsdlsl@naver.com (Y.C.); cooco526@nate.com (N.P.)

[2] School of Civil, Environmental and Chemical Engineering, Changwon National University, Changwon, Gyeongsangnamdo 51140, Korea

* Correspondence: jjh0208@changwon.ac.kr; Tel.: +82-552-133-748

Received: 15 November 2019; Accepted: 9 December 2019; Published: 12 December 2019

Supplementary Materials

Table 1. Ion source parameters.

**Ion source (HESI):**	
**Spray Voltage**	**3000 V**
Sheath Gas Flow	45 AU (Arbitrary Unit)
Aux Gas Flow	10 AU
Max Spray Current	100 A
Capillary Temp	320 °C
Probe Heater Temp.	50 °C
**Orbitrap:**	
Full Ms:	
Mass Range	100-1500 m/z
Mass Resolution	140’000
AGC Target	1,000’000
Maximal Injection Time	100 ms
Data Dependent MS2	
Mass Resolution	17’500
Microscan	1
AGC Target	500’000
Isolation window	1 m/z

Table 2. The LOD and LOQ of the target compounds (mg/kg).

	MI	CMI	BIT	DEP	DMP
LOD	0.005	0.02	0.005	0.005	0.02
LOQ	0.02	0.05	0.01	0.05	0.05

Table 3. Concentration of target compounds in CPs (mg/kg).

Products	MI	CMI	BIT	DEP	DMP
Shampoos					
SA1	N.D	N.D	N.D	<LOQ	<LOQ
SA2	N.D	N.D	N.D	N.D	0.26
SA3	N.D	N.D	N.D	0.4	N.D
SA4	N.D	N.D	N.D	N.D	N.D
SA5	N.D	N.D	N.D	N.D	N.D
SB1	N.D	N.D	N.D	N.D	N.D

SB2	N.D	N.D	N.D	N.D	N.D
SB3	N.D	N.D	N.D	N.D	N.D
SB4	N.D	N.D	N.D	N.D	N.D
SB5	N.D	N.D	N.D	N.D	N.D
SC1	N.D	N.D	0.165	N.D	N.D
SC2	N.D	N.D	N.D	N.D	N.D
Body Washer					
BWA1	<LOQ	N.D	N.D	N.D	N.D
BWA2	<LOQ	N.D	N.D	N.D	N.D
BWA3	<LOQ	N.D	N.D	N.D	N.D
BWA4	<LOQ	N.D	N.D	N.D	N.D
BWA5	<LOQ	N.D	N.D	N.D	N.D
BWB1	<LOQ	N.D	N.D	N.D	N.D
Face cleansers					
FCA1	N.D	N.D	N.D	N.D	N.D
FCA2	N.D	N.D	N.D	N.D	5.1
FCA3	N.D	N.D	N.D	N.D	7.9
FCA4	N.D	N.D	N.D	N.D	4.0
FCA5	N.D	N.D	N.D	N.D	4.1
FCB1	N.D	N.D	N.D	N.D	N.D
FCB2	N.D	N.D	N.D	N.D	N.D
FCB3	N.D	N.D	N.D	N.D	N.D
FCB4	N.D	N.D	N.D	N.D	N.D
FCB5	N.D	N.D	N.D	N.D	N.D
FCC1	N.D	N.D	N.D	N.D	N.D
FCC2	N.D	N.D	<LOQ	N.D	N.D
Lipstick					
LP1	N.D	N.D	N.D	1.1	10
LP2	N.D	N.D	N.D	<LOQ	12
LP3	N.D	N.D	N.D	N.D	N.D
LP4	N.D	N.D	N.D	N.D	21
LP5	N.D	N.D	N.D	N.D	1.1
Hair Dyes					
HD1	N.D	N.D	N.D	N.D	N.D
HD2	N.D	N.D	N.D	N.D	N.D
HD3	N.D	N.D	N.D	N.D	N.D
HD4	N.D	N.D	N.D	N.D	N.D
HD5	N.D	N.D	N.D	N.D	N.D
Dish Washer					
DWA1	N.D	N.D	N.D	N.D	N.D
DWA2	N.D	N.D	N.D	N.D	N.D
DWA3	N.D	N.D	N.D	N.D	N.D
DWA4	N.D	N.D	N.D	N.D	N.D
DWA5	N.D	N.D	N.D	N.D	N.D
DWB1	N.D	N.D	N.D	N.D	N.D
DWB2	N.D	N.D	N.D	2.0	N.D
DWB3	N.D	N.D	N.D	N.D	N.D
DWB4	N.D	N.D	N.D	N.D	N.D
DWB5	N.D	N.D	N.D	N.D	N.D
DWC1	N.D	N.D	N.D	N.D	N.D
DWC2	N.D	N.D	N.D	N.D	N.D
DWC3	N.D	N.D	N.D	N.D	N.D
DWC4	N.D	N.D	N.D	N.D	N.D
DWC5	N.D	N.D	N.D	N.D	N.D

Laundry detergents					
LDA1	N.D	N.D	103	N.D	N.D
LDA2	N.D	N.D	92	N.D	N.D
LDA3	N.D	N.D	103	N.D	N.D
LDA4	N.D	N.D	7.6	N.D	N.D
LDA5	N.D	N.D	49	N.D	N.D
LDB1	N.D	N.D	390	N.D	N.D
LDB2	N.D	N.D	509	N.D	N.D
LDB3	N.D	N.D	518	N.D	N.D
LDB4	N.D	N.D	419	N.D	N.D
LDB5	N.D	N.D	509	N.D	N.D
LDC1	N.D	N.D	125	N.D	N.D
LDC2	N.D	N.D	121	N.D	N.D
LDC3	N.D	N.D	78	N.D	N.D
LDC4	N.D	N.D	68	N.D	N.D
LDC5	N.D	N.D	1.2	N.D	N.D
Fabric Softener					
FSA1	1.1	1.47	49	1.1	N.D
FSA2	N.D	1.22	41	N.D	<LOQ
FSA3	N.D	1.37	41	N.D	<LOQ
FSA4	N.D	N.D	38	N.D	<LOQ
FSA5	N.D	N.D	39	N.D	<LOQ
FSB1	N.D	<LOQ	46	N.D	N.D
FSB2	N.D	<LOQ	44	N.D	N.D
FSB3	N.D	<LOQ	43	N.D	N.D
FSB4	N.D	<LOQ	48	N.D	N.D
FSB5	N.D	8.0	0.62	N.D	N.D
FSC1	N.D	N.D	N.D	N.D	N.D
FSC2	N.D	N.D	N.D	N.D	N.D
FSC3	N.D	N.D	N.D	N.D	N.D
FSC4	N.D	N.D	N.D	N.D	N.D
FSC5	N.D	N.D	N.D	N.D	N.D
